# Ensembl regulation resources

**DOI:** 10.1093/database/bav119

**Published:** 2016-02-16

**Authors:** Daniel R. Zerbino, Nathan Johnson, Thomas Juetteman, Dan Sheppard, Steven P. Wilder, Ilias Lavidas, Michael Nuhn, Emily Perry, Quentin Raffaillac-Desfosses, Daniel Sobral, Damian Keefe, Stefan Gräf, Ikhlak Ahmed, Rhoda Kinsella, Bethan Pritchard, Simon Brent, Ridwan Amode, Anne Parker, Steven Trevanion, Ewan Birney, Ian Dunham, Paul Flicek

**Affiliations:** ^1^European Molecular Biology Laboratory, European Bioinformatics Institute, Wellcome Trust Genome Campus, Hinxton, Cambridge CB10 1SD, UK; ^2^Wellcome Trust Sanger Institute, Wellcome Trust Genome Campus, Hinxton, Cambridge CB10 1SA, UK

## Abstract

New experimental techniques in epigenomics allow researchers to assay a diversity of highly dynamic features such as histone marks, DNA modifications or chromatin structure. The study of their fluctuations should provide insights into gene expression regulation, cell differentiation and disease. The Ensembl project collects and maintains the Ensembl regulation data resources on epigenetic marks, transcription factor binding and DNA methylation for human and mouse, as well as microarray probe mappings and annotations for a variety of chordate genomes. From this data, we produce a functional annotation of the regulatory elements along the human and mouse genomes with plans to expand to other species as data becomes available. Starting from well-studied cell lines, we will progressively expand our library of measurements to a greater variety of samples. Ensembl’s regulation resources provide a central and easy-to-query repository for reference epigenomes. As with all Ensembl data, it is freely available at http://www.ensembl.org, from the Perl and REST APIs and from the public Ensembl MySQL database server at ensembldb.ensembl.org.

Database URL: http://www.ensembl.org

## Introduction

In addition to providing long-term storage of genetic information across cell divisions, DNA is also a physical molecule with dynamic biochemical activity. Complex interactions with polymerases, transcription factors (TF) and enzymes that modify histones and DNA (1–3) as well as its spatial structure (4, 5) largely determine the functional activity of the cell’s chromatin, in particular the controlled transcription of genes (6), which in turn controls cell development (7). Variants on the active sites of these interactions, or regulatory elements (8), have been shown to be driving forces of evolution (9, 10) and disease (11).

Advances in laboratory assays have allowed us to measure this rich activity genome-wide. For example, histone modifications and TF binding locations previously identified with chromatin immunoprecipitation followed by microarray hybridisation (ChIP-chip) (12) now generally employ high-throughput sequencing (ChIP-seq) (13, 14); DNA methylation is assayed with MeDIP (15) or bisulphite sequencing (16); regions of open chromatin are identified with Formaldehyde-Assisted Isolation of Regulatory Elements (FAIRE) (17), DNase-seq (18) or ATAC-seq (19). These measurements can be used to identify regulatory elements (20–22), but also characterise disease (23). To detect any signal, it is crucial to survey many of these biochemical features, often running many assays on a considerable number of samples. For this reason, large consortia have already produced vast reference datasets (24–26).

To make sense of these large datasets, the Ensembl Regulation resources provide a rich and powerful framework to browse or query these data and enable cell types comparison. In addition to cell-type specific measurements, we provide a number of summaries, as well as mapping microarray probes to the current reference sequences. Alongside all other Ensembl resources (27–30), this data can be browsed on the web, but also accessed programmatically through MySQL, Perl or REST (31) for intensive queries. Finally, a BioMart server (32) allows users to extract the required data in bulk.

## Methods

### Uniform processing of epigenomic data

We first select cell types for which we have sufficient data to produce a segmentation (see below), and download all the epigenomic datasets associated with those cell types in the form of sequencing reads.

Since we are aggregating data from diverse sources, it is vital to remove artefacts due to differences in analysis pipelines. Moreover, scientific consortia generally have neither the remit nor the resources to update their analysis results each time the reference assembly or other genome annotation is updated. We therefore remap all of the original data onto the current reference genome, call peaks and normalise the signal with our uniform pipeline (33).

### Regulatory evidence

To assess the experimental evidence supporting the high level annotation, Ensembl’s regulation resources provide the underlying peaks and normalised sequence read coverage signals. This experimental data comes from various public datasets (see Table 1). We track its provenance and provide links to the raw data in primary database resources such as the European Nucleotide Archive (ENA) (34), ENCODE (26) or NCBI (25).

**Table 1.** Ensembl regulation experimental resources for release 77

Experimental resources						
Species	Data sets by				Feature types by class	Total features
	Source		Class			
Human	ENCODE:	**423**	Polymerase:	**23**	**2**	Experimental peaks: **11267991**
	Roadmap Epigenomics:	**62**	Histone mod:	**241**	**46**	
	Other:	**22**	TF:	**196**	**213**	Motif Features: **154884**
	Total:	**507**	Open Chromatin:	**25**	**2**	
Mouse	ENCODE:	**16**	Polymerase:	**5**	**1**	Experimental Peaks: **1016365**
	Other:	**57**	Histone mod:	**26**	**12**	
	Total:	**73**	TF:	**29**	**24**	Motif features: **126617**
			Open Chromatin:	**3**	**1**	

### Genome segmentation

Genome segmentation tools such as Segway (35) or ChromHMM (36) conveniently allow us to summarise multiple assays into a single annotation of the genome. Essentially, they cluster genomic regions by their associated experimental marks. They can thus replace multidimensional measurements with cluster identifiers, or states. For human, we provide Segway and ChromHMM segmentations of our data, in which we replace the state identifier numbers with more evocative functional labels: predicted promoter with TSS, predicted transcribed region, predicted promoter flank, predicted enhancer, CTCF enriched, predicted repressed, predicted low activity and predicted heterochromatin.

### The Ensembl regulatory build

**Figure 1.**
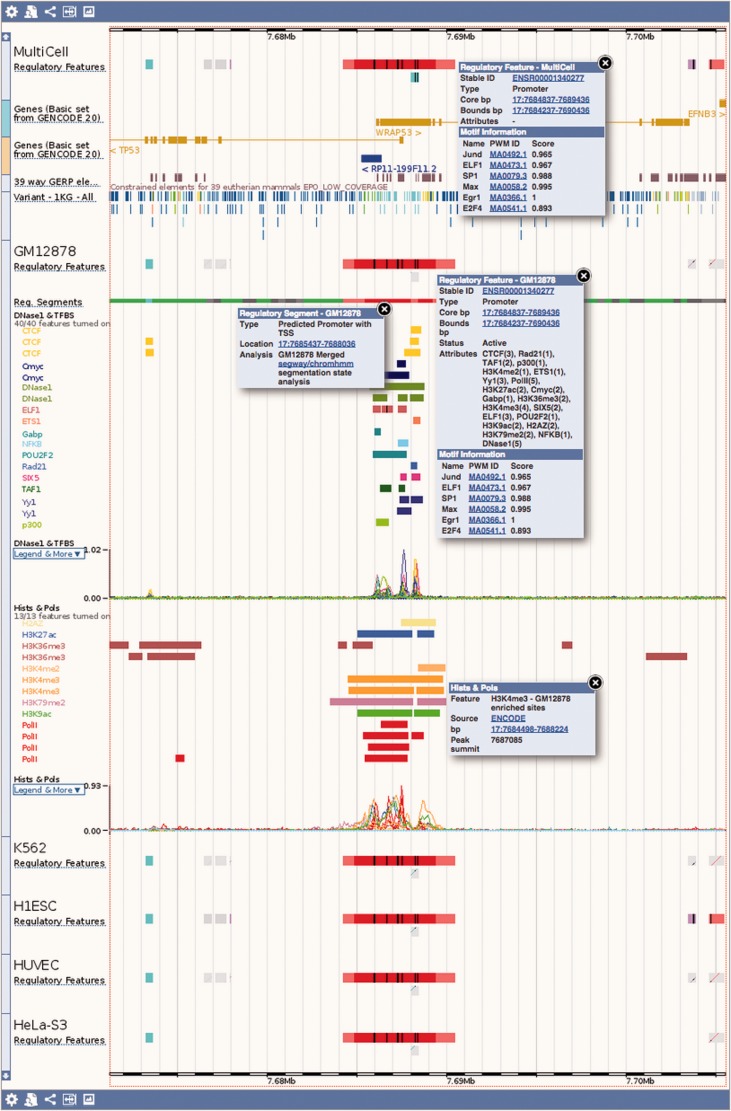
Regulation tracks. Screenshot of the ‘Region in detail’ location view, upstream of the human *TP53* gene. The default ‘MultiCell’ regulatory features track is shown. Below, the regulatory features, segmentation, ‘TFBS & DNase1’ and ‘Histones and Polymerases’ tracks associated to cell type *GM12878*. Finally, below again, the regulatory features annotated with activity for a few other cell types. Features that are inactive in a specific cell type are greyed out. Various elements were clicked to reveal floating menus with feature specific data and links to detailed views or external resources.

The Ensembl Regulatory Build has been redesigned to provide a straightforward summary of all the data processed above (33) (Figure 1). It is composed of consensus features (called MultiCell in the interface) that describe the function of a region. These functional labels are either inferred from the segmentation (promoter with TSS, promoter flanking region, distal enhancer or CTCF binding site) or directly from experimental measurements that are not explained by the segmentation (unannotated TF binding site–TFBS–or unannotated open chromatin). Each MultiCell feature is annotated with a cell type specific activity indicator (active or inactive).

### Motif features

Chromatin immunoprecipitation experiments locate genomic fragments bound by a protein. Nonetheless, they do not directly locate the exact TFBS at nucleotide resolution. We therefore annotate TF binding peaks with overlapping motif features, i.e. putative TFBS based on existing publicly TFBS Position Weight Matrices (PWM) from JASPAR (37).

We start by associating TFs used in ChIP experiments to Ensembl gene IDs based on the antibody used in the ChIP experiment. We then associate each JASPAR matrix to Ensembl genes. If the JASPAR protein identifier is from the correct species, this is done directly using Ensembl’s database of cross-linked identifiers across bioinformatics resources. Otherwise, the matrix is associated to the Ensembl human gene with the best Blastp (38) alignment to the JASPAR protein (blastp version: 2.2.15, parameters: -M BLOSUM80 -m 8 -b 1 –i). This process allows us to use any metazoan PWM for human and maximise the number of TFs covered.

To annotate TFBSs, we start by taking all JASPAR TF PWMs and do a lenient whole genome search for motif matches using MOODS (39). We then select all the matches that lie within observed ChIP-seq binding regions. We discard any match whose score has a single tail p-value above 5%, as estimated empirically from the distribution of scores obtained by selecting matches on random regions of the genome. Finally, we associate filtered matches to overlapping experimental features and their associated regulatory feature.

Of the 211 human TFs for which we have experimental data, 45 have at least one associated PWM (in total, 97 PWMs are associated, see Supplementary Table 1). Ensembl release 77 (October 2014) includes 154 884 distinct TFBS for human. Similarly, of the 23 mouse TFs for which we have experimental data, 12 have an associated PWM (19 PWMs in total, see Supplementary Table 2). They are associated to 126 617 TFBS. Note that not all TFs have an associated PWM in JASPAR. The fraction of peaks with an associated motif hit, assuming a PWM is known, is highly variable (see table S3), however for strong binding pioneer TFs such as CTCF a PWM is found at a substantial majority of binding sites.

### DNA methylation

**Figure 2.**
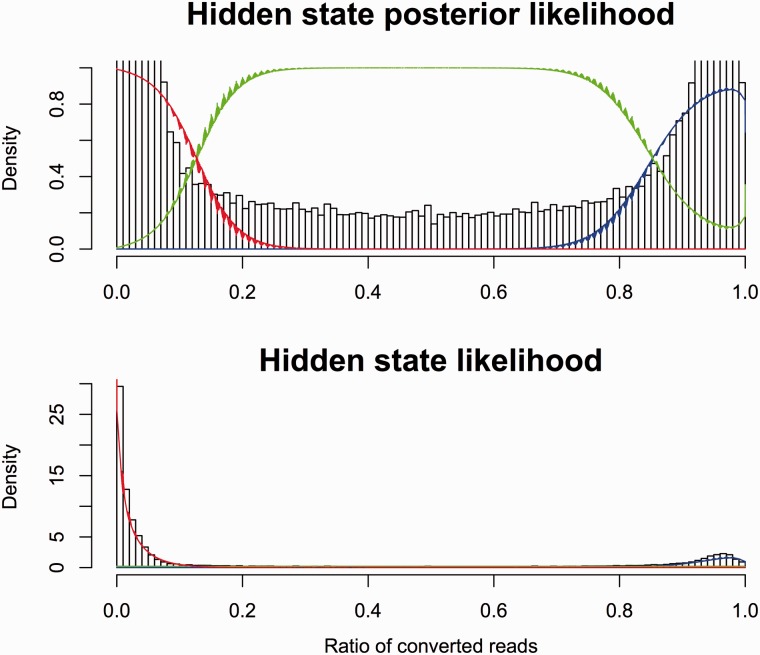
Modelling bisulphite sequencing results with a Bayesian model. The fraction of converted reads in bisulphite sequencing is correlated to the methylation status of the underlying DNA. We show here a Bayesian model that fits the observed data by dividing the cytosines into three categories: methylated (red), unmethylated (blue) and unknown (green).

Both Reduced Representation and Whole Genome Bisulfite Sequencing (RRBS/WGBS) data (16, 25, 26, 40) are provided via the database, characterising 44 cell lines. This data was processed with Bismark (41) with default parameters, and replicate counts were merged. The number of converted reads at each position was fitted to a mixture model of two beta-binomial distributions and a uniform discrete distribution conditioned on the total number of reads at that position. This model assumes that a base pair is fully methylated, fully unmethylated or in an undetermined third state. For each dataset, the parameters of these distributions were set by Expectation Maximization (EM) across all the data points. See Figure 2 for an example fit. Using these parameters, we evaluated the posterior probability of all three states at each observed position. If all three states had a posterior probability >10^−^^4^, then the position was discarded.

### Externally curated data

Ensembl stores a small set of externally curated datasets pertinent to gene regulation. These include: a set of human and mouse miRNA target predictions provided by DIANA-TarBase (42), experimentally validated non-coding regions from the VISTA enhancer browser (43), and regions highlighted by the FANTOM 5 project (44, 45).

### Microarray probe mappings

Microarray probe mappings are provided for widely used microarray platforms across several species. See Supplementary Table S4 for a summary of the supported platforms. These are primarily expression arrays, with a smaller number of human methylation and comparative genomic hybridization (CGH) arrays. Represented manufacturers include Affymetrix, Illumina, Codelink, Agilent and Phalanx, as well as small number of custom array designs. Our methods for mapping probes and the resulting annotations were described in Ballester *et al.* (46).

### Experimental metadata

Normalised annotation of experimental metadata (such as the cell type and experimental factor) is essential for data integration. Large-scale projects such as ENCODE have led the way by using internal stable nomenclatures. To improve traceability and data integration, we use the Experimental Factor Ontology (EFO) (47), and actively work with the EFO curation team to correct or submit new entries as required.

### Data visualisation and access

#### Ensembl location view

Ensembl regulation data can be visualised in the Ensembl genome browser (48). Figure 1 shows an example of the main Location view, where regulatory features can be observed alongside gene models and other user-selected data. Clicking on a regulatory feature reveals a pop-up menu with basic details: feature coordinates, stable ID, its classification and mapped TF binding motifs. By default, only the MultiCell regulatory features are displayed in this view, but the display can configured to show additional cell-specific annotations and regulatory evidence using the configuration panel linked on the left hand side of the page.

Experimental evidence for the MultiCell regulatory features can be displayed as regions, generally ChIP-seq peak calls. Clicking on a peak displays its type (e.g. DNase1 or the specific TF name); the parameters that defined the region from the data including score and peak summit representing the position of highest sequencing read coverage; and a data source such as an ENA accession. Additionally, the read coverage density functions underlying the peaks, measured in Reads Per Kilobase per Million mapped reads (RPKM), can be displayed as curves.

Motif features (PWM alignments) are displayed as black boxes within regulatory features and ChIP-seq peaks of the corresponding TF. Clicking on a motif feature reveals a floating menu where the TF name, JASPAR PWM ID and the ratio of the motif’s binding score divided by the PWM’s optimal binding affinity are all highlighted within a Motif Information table.

The regulatory segmentation data is shown as a continuous line across the genome whose colour changes with the local classification. Clicking on a segment reveals its classification, its exact genomic location, and the analysis used to generate the segmentation.

DNA CpG methylation can be displayed as markers, whose color moves along a gradient from yellow (hypo-methylated) through to green then blue (hyper-methylated). A click reveals the methylation information for any given cytosine, when zoomed out these changes to providing summary information for the selected region.

Other externally curated sets of functional elements (expression quantitative trait locus–eQTL–regions, miRNA targets and VISTA regions) can also be displayed as feature tracks, with appropriately detailed feature menus.

#### Configuring the location view

**Figure 3.**
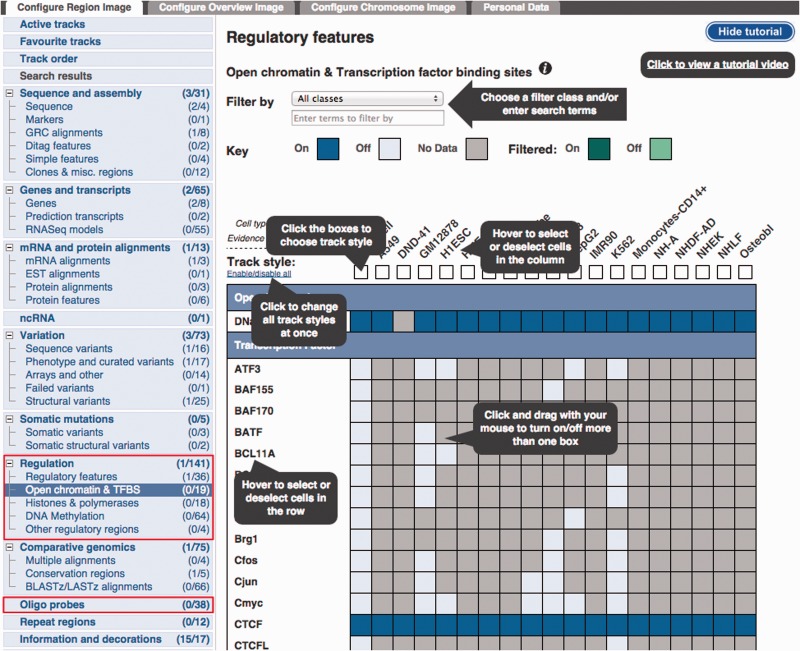
Configuration panel and data selection matrix. This panel is available via the ‘Configure this page’ button in the location view. The ‘Regulation’ menu items describe the different types of data available. Lower down, the ‘Oligo Probes’ menu items provides access to microarray probe mappings. The ‘Open chromatin & TFBS’ item has been selected to reveal a configuration matrix. To assist first time users, a tutorial is presented, this tutorial can be hidden or shown as desired.

The Regulation tracks can be configured in the ‘Regulation’ section of the display configuration panel (Figure 3). The submenu items restrict the panel to subsets of available tracks.

**Figure 4.**
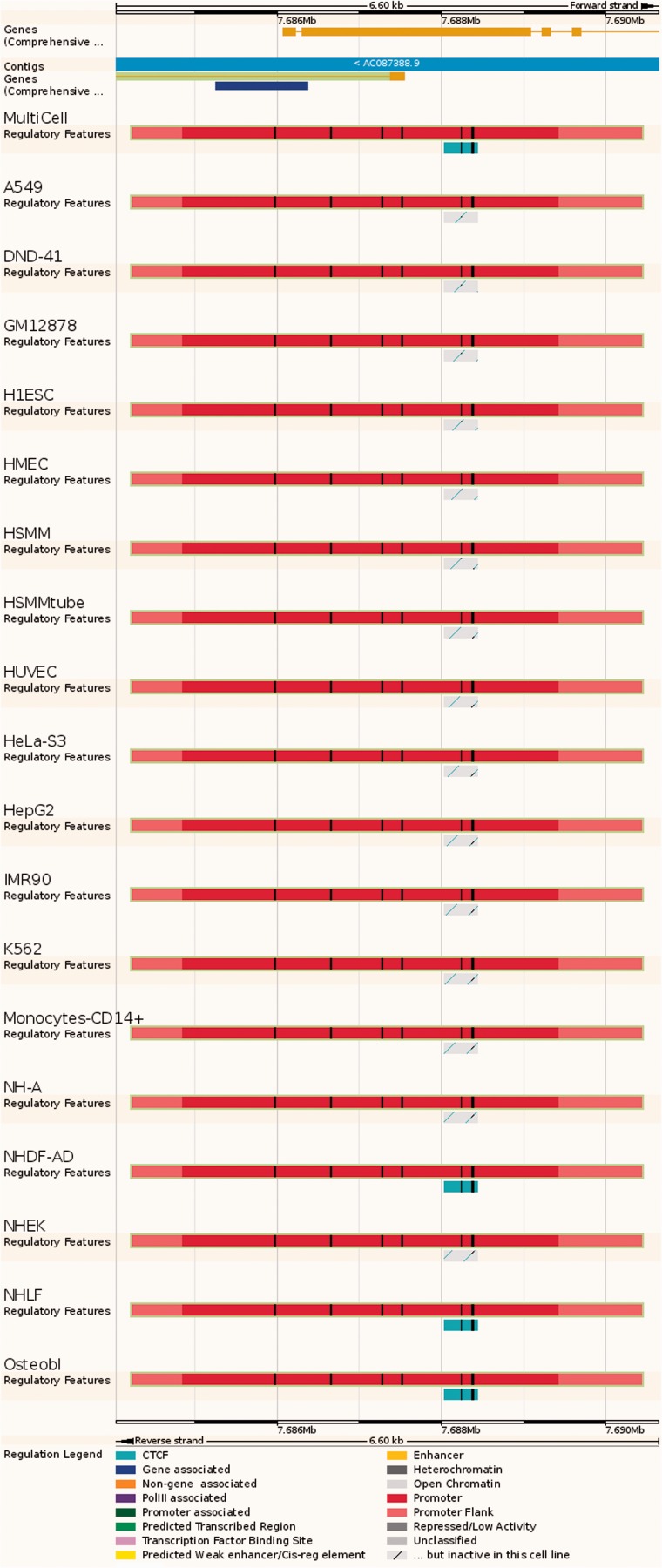
Regulatory Feature ‘Summary’ view. This view displays the genes overlapped by the regulatory element of interest, along with activity information in all available cell types. In this case, the red promoter is constitutively active, but the nearby cyan CTCF binding site is active in only 4 cell types.

**Figure 5.**
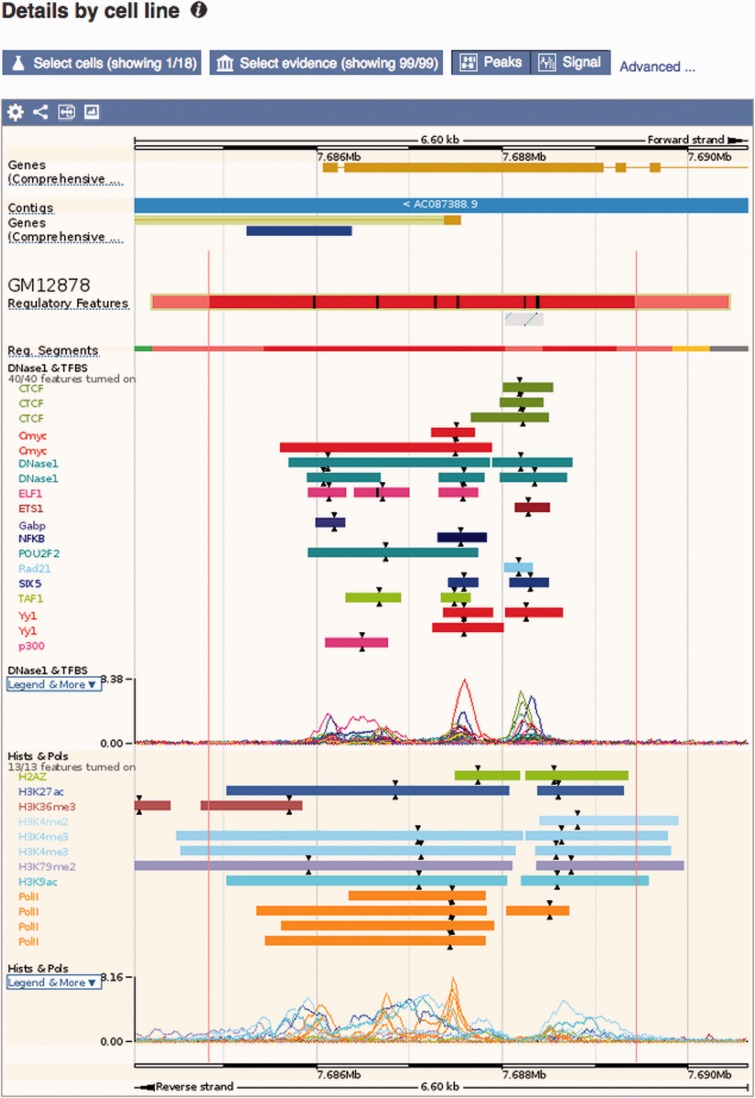
Regulatory feature ‘Details by cell line’ view. This view displays all the evidence surrounding a regulatory feature of interest, either as regions (ChIP-seq peaks) or signal functions. At the top, a solid multicolored bar represents the segmentation for the desired cell types. Note the selector buttons above the window.

**Figure 6.**
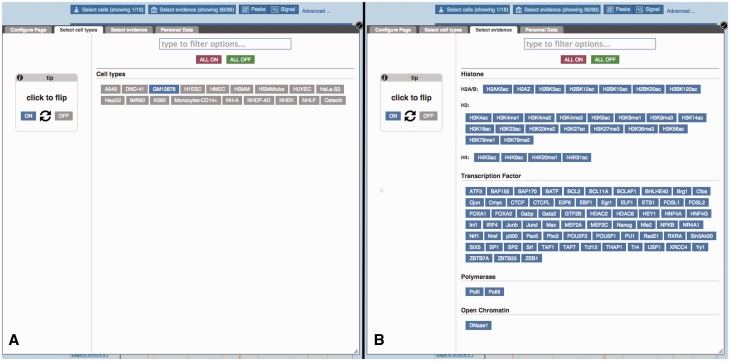
Regulatory Feature ‘Details by cell line’, cell type and experimental evidence selectors. (**A**) At the top of the ‘Details by cell line view’ (Figure 5) a button labelled ‘Select cells’ displays this modal window, which allows the user to quickly toggle cell types on or off. (**B**) Similarly, the ‘Select evidence’ button displays this modal window, which allows the user to quickly toggle experimental tracks on or off.

Because of the large number of available cell types and experiments, many of the experimental tracks are organised in tables where each column is specific to a cell type and each row to a type of assay. A search box facilitates filtering the list of available factors. Clicking or dragging the mouse selects single or multiple tracks. Pointing the mouse above an axis label reveals a button to select or deselect all the cells on that row or column. For each column, the user can choose between displaying regions (peaks), or plots of sequencing read coverage depth (signal).

The display of microarray probe mappings can be configured from the top-level ‘Oligo Probe’ section in the configuration menu.

### Inspecting a regulatory feature

**Figure 7.**
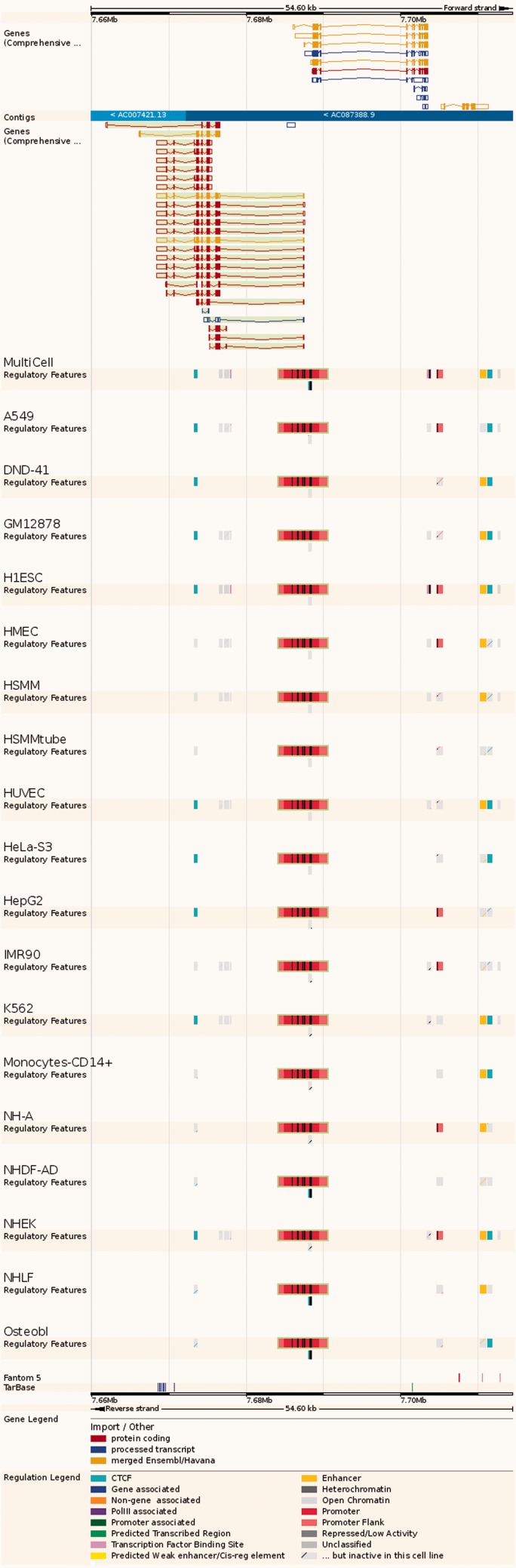
Regulatory Feature ‘Feature context view’. This view displays the general context 25 kb upstream and downstream of a feature, with genes and neighbouring elements. As in the summary view, for each cell type inactive features are greyed out, with sparse coloured lines indicating their underlying function. At the bottom, FANTOM5 elements and TarBase target regions are indicated.

Clicking on a regulatory feature stable ID leads to a ‘Regulation’ tab, where more detailed information is displayed about that feature, including cell-specific annotations and supporting regulatory evidence that are usually not displayed in the Location view. On the top of the screen, large icons lead to different displays. Each display starts with the location of the feature and a summary of its activity in each cell type, followed by a specialised view:

**Summary:** a lightweight view that displays the 'MultiCell' regulatory feature and its activity in different cell types (see Figure 4).
**Details by cell line**: Cell-specific features are shown alongside their supporting evidence (Figure 5). To aid interpretation, vertical red lines delineate the core region of the selected regulatory feature. Buttons at the top of the display bring up modal windows to quickly toggle cell types and experimental evidence on or off (see Figure 6).
**Feature Context:** A display of wider genomic context around the chosen regulatory feature (see Figure 7).
**Evidence:** A tabular view displaying the list of all supporting evidence peaks for that regulatory feature. The table header allows the user to filter and export the table.

### Example use case

The applications of this regulatory annotation are expected to expand with time. Key current uses include prioritising regulatory variants and observing the dynamics of gene expression regulation. For example, oestrogen receptor gene ESR1 is known to have an alternate distal promoter in osteoblasts and some cancer cell lines (49). This alternate promoter is clearly identified in the Regulatory Build, as shown in Figure S5.

### Experimental Meta-data

Tracking the origin of experimental evidence is essential for traceability and reproducibility. We store experimental meta-data in the Ensembl ‘Funcgen’ database, and these data can be accessed through the browser. Selecting an experimental peak reveals a link to the original data. This link is labelled with either a sequence archive ID (referring to the exact set of sequencing reads used in the Ensembl analysis) or a project name such as ENCODE if the data has not yet been submitted or deposited in an archive. Selecting this link leads to an Experiment view, containing a summary table of all available experiments, and a more detailed Sources table describing the sources of the selected feature. The summary table can be used to filter the content of the detailed Sources panel, allowing easy access and comparison of the meta-data for all the experiments incorporated into the Regulatory Build. By clicking links in the summary table, filters on cell type, project name and evidence type can be added or removed. The system automatically composes the filters to display the desired datasets. In the Sources table, the ‘source’ and ‘project’ fields link out to the location of the source data (either in a sequence archive or on an associated project page) and the project website respectively. Other fields here include evidence type and name, links to the cell type EFO definition, PWMs and associated encoding genes for TF experiments. More fine-grained filtering is again enabled in the table header search box, allowing searches for more specific feature types.

### BioMart

**Figure 8.**
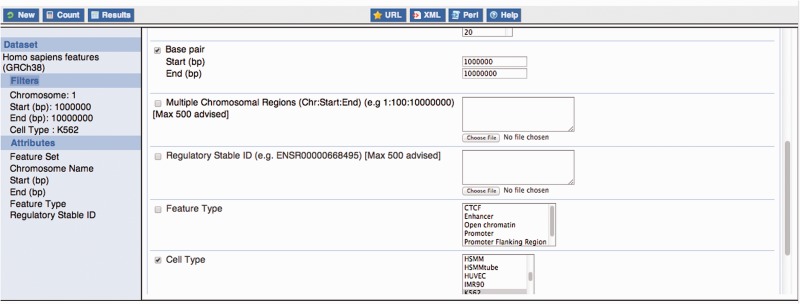
Constructing a BioMart query. This figure exemplifies the construction of a BioMart query to obtain all regulatory features for K562, within the region 1:1000 000–10 000 000 and display a varied number of properties for those regulatory regions, including their stable ids.

Large-scale querying of Ensembl regulation data is possible through the Ensembl BioMart interface (32), which can output large numbers of features in several formats including tab-delimited text. Using the ‘Ensembl Regulation’ database (see Figure 8) as a starting point the user can select a regulation data set for a given species. The human and mouse data sets contain regulatory features, as well as regulatory evidence (experimental peaks), binding motifs (TFBS PWM mappings) and other regulatory regions (externally curated data). The *Drosophila* data set currently contains the latter only.

Using filters, data for specific cell lines and/or specific experimental factors can be selected as can regulatory features overlapping specific genomic regions (e.g. upstream genes of interest).

Microarray probe mappings and transcript annotations can be obtained through the ‘Ensembl Genes’ database, by selecting the relevant array related filters or external attributes. Methylation data are not currently available through BioMart.

### Within the Variant Effect Predictor

**Figure 9.**
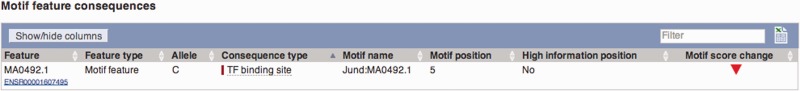
VEP output using motif feature information. Subsection of the VEP report on SNP rs694061. The VEP reports whether a SNP affects the binding affinity of a motif feature (i.e. whether position of the SNP contains at least 1.5 bits of information on the PWM).

The Ensembl Variant Effect Predictor (VEP) (50) has recently added regulatory features to its set of predicted consequences that can be associated with single nucleotide polymorphisms (SNP) or *de novo* variants. This provides an additional source of evidence for the effect of variants that, although significantly associated to diseases or other phenotypes, have currently little functional annotation since they often fall outside known coding regions. The VEP also reports whether a variant falls in a TFBS binding motif and, in the case of SNPs, whether it lies in a highly informative position of the TFBS, with potentially significant effects on the TF binding affinity (see Figure 9).

### Programmatic data access

As with other Ensembl resources, regulation data can be extracted using a specific Perl Application Programming Interface (API) or by directly accessing the public funcgen MySQL databases at ensembldb.ensembl.org. The Ensembl website hosts specific funcgen tutorials and documentation describing this access (http://www.ensembl.org/info/docs/funcgen/index.html). For other programming languages, key extraction functions are offered via the RESTful interface (31).

### Data downloads

Ensembl regulation data is also available for download on the FTP site (http://www.ensembl.org/info/data/ftp/index.html). This includes separate GFF files for each cell specific and MultiCell Regulatory Build (RegulatoryFeatures), as well as supporting experimental evidence (Annotated Features) and PWM alignments (MotifFeatures).

## Discussion

The Ensembl regulation resources will continue to grow and evolve, accompanying the fields of genome regulation and epigenomics as new results are produced and new experimental techniques and analyses are developed. Firstly, in terms of scale, we expect the number of available cell types to climb rapidly to the hundreds, as more groups are contributing epigenomes to the global research community via the International Human Epigenome Consortium (IHEC) and other efforts. In particular, tissue samples will progressively replace the cell line models used thus far, which will enable us to detect regulatory features with greater sensitivity, since we are likely missing a considerable number of dynamic tissue-specific elements such as enhancers.

Since our focus is to provide complete cell type specific annotations, we cover only chosen cell types, and do not attempt to include all publicly available epigenomic datasets. These data can nonetheless be visualised in Ensembl using, for example, the IHEC data portal’s track hub function (http://epigenomesportal.ca/ihec/).

Our limited knowledge of TF binding motifs will need to be addressed. Current public databases such as JASPAR (37) only cover a fraction of existing TFs. Other techniques such as UniPROBE (51) or SELEX (52) will allow us to describe far richer binding interactions at enhancer and promoter sites.

New technologies, such as chromatin conformation capture (53–55) will add a spatial component to our datasets. This might in turn affect analysis tools, such as segmentation, which could take into account spatial proximity when computing state likelihoods (56).

There will also be attempts to describe the cis- interactions of the regulatory elements thus uncovered. Already, signal correlation (43, 57), eQTLS (58, 59) and chromatin conformation capture (60) are being improved to eventually produce a map of the regulatory network of the genome. As these approaches are refined, they will significantly enrich the annotations of the features defined by the Ensembl Regulatory Build.

## Conclusion

Ensembl’s regulation resources aim to document all the available knowledge on gene expression regulation and epigenomics. We are progressively accumulating data on as many cell types as possible, describing histone marks, transcription factor binding, DNA modifications and transcription factor binding motifs. Much like the research field it is serving, this resource is still in an expansion phase and resolutely turned towards new experimental techniques.

## Availability

All Ensembl data and source code are freely available and may be downloaded in their entirety from the Ensembl website. Each Ensembl release is maintained as an archive web site for at least 3 years after the date of initial release. Ensembl is updated approximately five times each year with new data, genome assemblies, and sequenced genomes. Additionally, the data is available through a programmatic interface and through the web-based Ensembl Biomart.

## Supplementary Material

Supplementary Data
