# Supplementary material for: Ensembl regulation resources
Source: Database (Oxford). 2016 Feb 16;2016:bav119. doi: 10.1093/database/bav119 (PMC4756621; doi:10.1093/database/bav119)
Supplement: Supplementary Data [file supp_2016_bav119_index.html]

Ensembl regulation resources — Supplementary Data 

# Ensembl regulation resources

## Supplementary Data

files

- Supplementary Data - docx file
