# Supplementary material for: Ensembl regulation resources
Source: Database (Oxford). 2016 Feb 16;2016:bav119. doi: 10.1093/database/bav119 (PMC4756621; doi:10.1093/database/bav119)
Supplement: Supplementary Data [file supp_bav119_Supplemental_Methods.docx]

# Supplemental Materials to:

# Ensembl Regulation Resources

Daniel R Zerbino, Nathan Johnson, Thomas Juetteman, Dan Sheppard, Steven P Wilder, Ilias Lavidas, Michael Nuhn, Emily Perry, Quentin Raffaillac-Desfosses, Daniel Sobral, Damian Keefe, Stefan Gräf, Ikhlak Ahmed, Rhoda Kinsella, Bethan Pritchard, Simon Brent, Ridwan Amode, Anne Parker, Steven Trevanion, Ewan Birney, Ian Dunham and Paul Flicek

## Supplementary Tables

### Table S1 – Mapping Jaspar Matrices to Human Ensembl IDs

| **TF** | **Ensembl Gene** | **JASPAR motifs** |
| --- | --- | --- |
| ZNF263 | ENSG00000006194 | MA0528.1 |
| POU2F2 | ENSG00000028277 | MA0507.1,PH0144.1 |
| MEF2A | ENSG00000068305 | MA0005.2,MA0052.2,MA0558.1,MA0563.1,MA0585.1 |
| FOSL2 | ENSG00000075426 | MA0478.1 |
| MEF2C | ENSG00000081189 | MA0005.1,MA0497.1,MA0555.1,MA0559.1 |
| REST | ENSG00000084093 | MA0138.1,MA0138.2 |
| YY1 | ENSG00000100811 | MA0095.1,MA0095.2 |
| HNF4A | ENSG00000101076 | MA0114.1,MA0114.2,PB0030.1,PB0134.1 |
| GATA1 | ENSG00000102145 | MA0035.1,MA0035.2,MA0035.3,MA0293.1 |
| CTCF | ENSG00000102974 | MA0139.1 |
| NRF1 | ENSG00000106459 | MA0506.1 |
| VDR | ENSG00000111424 | MA0016.1,MA0065.2,MA0159.1,MA0494.1,MA0512.1,MA0534.1,PB0057.1,PB0161.1 |
| SRF | ENSG00000112658 | MA0083.1,MA0083.2,MA0271.1,MA0331.1,PB0078.1 |
| ELF1 | ENSG00000120690 | MA0473.1 |
| EGR1 | ENSG00000120738 | MA0162.1,MA0162.2,MA0337.1,MA0341.1,MA0366.1,MA0423.1,PB0010.1,PB0114.1 |
| CTCFL | ENSG00000124092 | MA0531.1 |
| FOXA2 | ENSG00000125798 | MA0047.1,MA0047.2,MA0446.1,PB0015.1 |
| MAX | ENSG00000125952 | MA0058.1,MA0058.2,MA0059.1,PB0043.1,PB0147.1,PL0007.1 |
| FOXA1 | ENSG00000129514 | MA0148.1,MA0148.3,MA0546.1 |
| JUND | ENSG00000130522 | MA0491.1,MA0492.1 |
| NR1H2 | ENSG00000131408 | MA0016.1,MA0065.2,MA0159.1,MA0494.1,MA0512.1,MA0534.1,PB0057.1,PB0161.1 |
| THAP1 | ENSG00000131931 | MA0597.1 |
| PPARG | ENSG00000132170 | MA0016.1,MA0065.2,MA0159.1,MA0494.1,MA0512.1,MA0534.1,PB0057.1,PB0161.1 |
| BHLHE40 | ENSG00000134107 | MA0464.1,PB0007.1,PB0111.1 |
| ETS1 | ENSG00000134954 | MA0098.2 |
| MYC | ENSG00000136997 | MA0058.1,MA0058.2,MA0059.1,PB0043.1,PB0147.1,PL0007.1 |
| IRF4 | ENSG00000137265 | PB0034.1,PB0138.1 |
| TCF12 | ENSG00000140262 | MA0521.1 |
| ZEB1 | ENSG00000148516 | MA0103.2 |
| USF1 | ENSG00000158773 | MA0093.1,MA0093.2,MA0281.1 |
| TAL1 | ENSG00000162367 | MA0035.1,MA0035.2,MA0035.3,MA0293.1 |
| EBF1 | ENSG00000164330 | MA0154.2 |
| HNF4G | ENSG00000164749 | MA0484.1 |
| PBX3 | ENSG00000167081 | MA0318.1,MA0328.1 |
| SP2 | ENSG00000167182 | MA0516.1 |
| E2F6 | ENSG00000169016 | MA0471.1 |
| JUNB | ENSG00000171223 | MA0490.1 |
| FOSL1 | ENSG00000175592 | MA0477.1 |
| NR2C2 | ENSG00000177463 | MA0504.1 |
| ZBTB33 | ENSG00000177485 | MA0527.1 |
| GATA2 | ENSG00000179348 | MA0036.1,MA0036.2 |
| SP1 | ENSG00000185591 | MA0079.1,MA0079.2,MA0079.3 |
| RXRA | ENSG00000186350 | MA0016.1,MA0065.2,MA0159.1,MA0494.1,MA0512.1,MA0534.1,PB0057.1,PB0161.1 |
| PAX5 | ENSG00000196092 | MA0014.1,MA0014.2,MA0239.1 |
| E2F4 | ENSG00000205250 | MA0470.1,MA0541.1 |

### Table S2 – Mapping Jaspar Matrices to Mouse Ensembl IDs

| **TF** | **Ensembl gene** | **Jaspar Matrix** |
| --- | --- | --- |
| Klf4 | ENSMUSG00000003032 | MA0039.1,MA0039.2 |
| Stat3 | ENSMUSG00000004040 | MA0144.1 |
| Ctcf | ENSMUSG00000005698 | MA0139.1 |
| Myb | ENSMUSG00000019982 | MA0100.1,PB0045.1,PB0149.1 |
| Esrrb | ENSMUSG00000021255 | MA0141.1 |
| Myc | ENSMUSG00000022346 | MA0059.1,MA0147.1 |
| Tfcp2l1 | ENSMUSG00000026380 | MA0145.1 |
| E2f1 | ENSMUSG00000027490 | MA0024.1 |
| Mycn | ENSMUSG00000037169 | MA0104.1,MA0104.2 |
| Max | ENSMUSG00000059436 | PB0043.1,PB0147.1,PL0007.1 |
| Sox2 | ENSMUSG00000074637 | MA0143.1 |
| Zfx | ENSMUSG00000079509 | MA0146.1 |

**Table S3: Coverage of ChIP-Seq peaks by recognizable PWM matches:**

| TF | # ChIP-Seq peaks | Fraction of peaks with significant PWM matches |
| --- | --- | --- |
| Egr1 | 23095 | 0.9404 |
| Nrf1 | 877 | 0.9259 |
| E2F4 | 3175 | 0.8813 |
| USF1 | 30053 | 0.8766 |
| PU1 | 15256 | 0.8577 |
| FOSL2 | 3629 | 0.844 |
| CTCF | 1020553 | 0.7847 |
| Cjun | 31384 | 0.7517 |
| Gabp | 22886 | 0.7517 |
| Jund | 20132 | 0.7263 |
| Junb | 441 | 0.7143 |
| ZBTB33 | 2336 | 0.714 |
| RXRA | 1484 | 0.6941 |
| FOSL1 | 2128 | 0.6786 |
| Srf | 5822 | 0.6539 |
| NFKB | 914 | 0.651 |
| HNF4A | 8515 | 0.6377 |
| SP2 | 919 | 0.6159 |
| CTCFL | 1775 | 0.6096 |
| Nrsf | 24940 | 0.6021 |
| HNF4G | 1110 | 0.5838 |
| FOXA2 | 1815 | 0.5405 |
| EBF1 | 5360 | 0.5187 |
| Gata1 | 798 | 0.4812 |
| MEF2C | 234 | 0.4786 |
| MEF2A | 5051 | 0.474 |
| SP1 | 9544 | 0.4416 |
| FOXA1 | 16490 | 0.4115 |
| ZEB1 | 605 | 0.4017 |
| Yy1 | 37840 | 0.3932 |
| Cfos | 6879 | 0.3649 |
| ELF1 | 13995 | 0.3645 |
| Znf263 | 2721 | 0.3602 |
| E2F6 | 10436 | 0.3595 |
| BHLHE40 | 198 | 0.3535 |
| Pax5 | 2232 | 0.3477 |
| POU2F2 | 790 | 0.3114 |
| Cmyc | 33983 | 0.2714 |
| Tcf12 | 6241 | 0.2674 |
| Gata2 | 4216 | 0.2308 |
| THAP1 | 651 | 0.2243 |
| Tr4 | 2248 | 0.2211 |
| IRF4 | 3127 | 0.2194 |
| ETS1 | 1337 | 0.1488 |
| Max | 47936 | 0.0988 |
| Pbx3 | 1097 | 0.0273 |

**Table S4: Microarray probesets stored in Ensembl Regulation**

| Species | Vendors | Arrays | Annotated Probe(set)s | % Annotated Probe(set)s | Annotated transcripts | % Annotated transcripts |
| --- | --- | --- | --- | --- | --- | --- |
| *Bos taurus* | 1 | 1 | 22958 | 95% | 11281 | 42% |
| *Caenorhabditis elegans* | 5 | 10 | 212557 | 81% | 26979 | 47% |
| *Canis familiaris* | 1 | 1 | 42012 | 98% | 22963 | 77% |
| *Ciona intestinalis* | 1 | 1 | 30576 | 99% | 12081 | 68% |
| *Danio rerio* | 4 | 7 | 62060 | 68% | 16660 | 29% |
| *Drosophila melanogaster* | 1 | 2 | 32740 | 99% | 26425 | 91% |
| *Gallus gallus* | 1 | 1 | 36672 | 95% | 14205 | 79% |
| *Homo sapiens* | 5 | 33 | 2483116 | 89% | 190735 | 92% |
| *Macaca mulatta* | 1 | 1 | 51669 | 98% | 26450 | 59% |
| *Mus musculus* | 5 | 22 | 1507958 | 91% | 95325 | 95% |
| *Ornithorhynchus anatinus* | 1 | 1 | 23824 | 100% | 25380 | 91% |
| *Oryctolagus cuniculus* | 1 | 2 | 46115 | 54% | N/A | N/A |
| *Pan troglodytes* | 1 | 16 | 1487689 | 90% | 25931 | 89% |
| *Rattus norvegicus* | 5 | 16 | 1239056 | 93% | 27669 | 95% |
| *Saccharomyces cerevisiae* | 1 | 2 | 15039 | 74% | 6836 | 96% |
| *Sus scrofa* | 1 | 1 | 22050 | 91% | 12127 | 40% |
| *Xenopus tropicalis* | 1 | 1 | 52673 | 89% | 18872 | 78% |

**Figure S5: Examining *ESR1* promoter activity across cell types.**

1. We were able to confirm through the Ensembl Regulatory Build an observation made in the literature (52), namely that the distal promoter of *ESR1* is active in Osteoblasts and some cancer cells. This is visible within the vertical highlighted rectangle, where the promoter is generally inactive (grey) but active (red) in Osteoblasts and HSMM, HSMMtube, NHDF-AD (a fibroblast cancer cell line). HSMM and HSMMtube, two cultures derived from human skeletal muscle cells and myoblasts do not fit the model, but the consistency between the two related cell types suggests the measurement is correct.

**
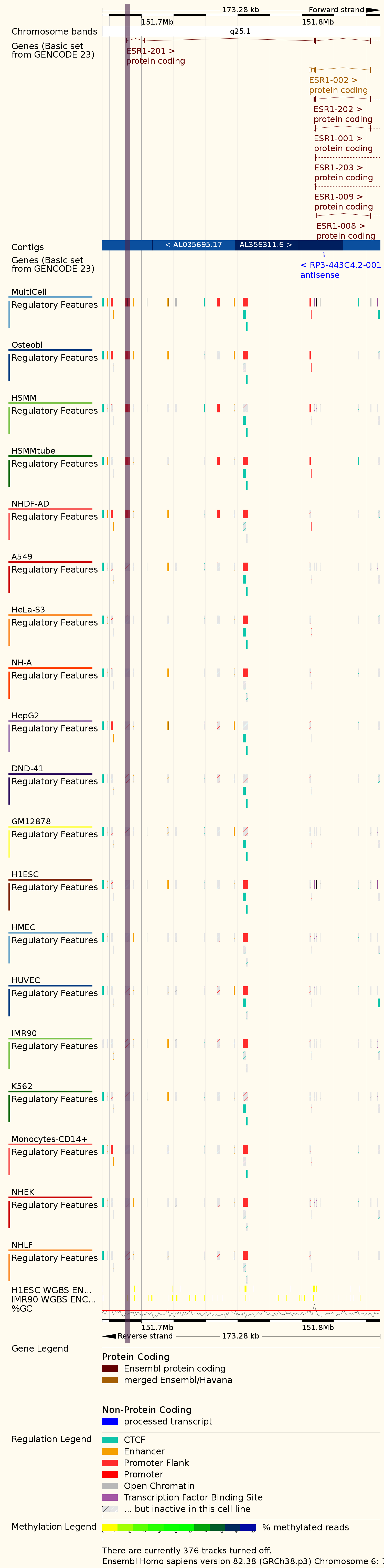
**
